# Intraoperative rotational kinematics and its influence on postoperative clinical outcomes differ according to age in Unicompartmental knee Arthroplasty

**DOI:** 10.1186/s12891-021-04371-w

**Published:** 2021-06-01

**Authors:** Kohei Kawaguchi, Hiroshi Inui, Shuji Taketomi, Ryota Yamagami, Kenichi Kono, Shin Sameshima, Tomofumi Kage, Sakae Tanaka

**Affiliations:** Department of Orthopaedic Surgery, Faculty of Medicine, The University of Tokyo, 7-3-1 Hongo, Bunkyo-ku, Tokyo, 113-0033 Japan

**Keywords:** Unicompartmental arthroplasty, Elderly patients, Patient-reported outcome, Intraoperative kinematics, Navigation

## Abstract

**Background:**

Although Oxford unicompartmental knee arthroplasty (UKA) is used in patients of wide age ranges, there is no clear information regarding the age differences in terms of intraoperative femorotibial rotational kinematics and its influence on clinical outcomes. Therefore, this study was conducted to examine the age differences in terms of intraoperative rotational kinematics and postoperative clinical outcomes and to analyze their relationship with classification according to the age group.

**Methods:**

We investigated 111 knees of patients who underwent Oxford UKA using a navigation system and divided them into two groups: elderly (aged ≥75 years; 48 knees) and nonelderly (aged < 75 years; 63 knees). Intraoperative tibial internal rotational angles relative to the femur during passive knee flexion were measured using a navigation system, and clinical outcomes were evaluated using knee range of motion, the Knee Injury and Osteoarthritis Outcome Score (KOOS), and the Knee Society Functional Score at 2 years postoperatively. The relationships between intraoperative tibiofemoral rotational angles and clinical outcomes were also evaluated in the two groups.

**Results:**

The intraoperative tibial internal rotational angle relative to the femur during knee flexion was significantly larger in the nonelderly group (13.5°) than in the elderly group (9.0°). The intraoperative tibial internal rotational angle showed a positive correlation with the pain subscale of KOOS only in the nonelderly group.

**Conclusion:**

Intraoperative rotational kinematics and its influence on clinical outcomes were different between elderly and nonelderly patients, and the tibial internal rotational angle could be a more important factor for successful UKA in nonelderly patients.

## Background

Mobile-bearing Oxford unicompartmental knee arthroplasty (UKA) (Zimmer Biomet Ltd., Swindon, UK) has been used successfully for more than 30 years to treat anteromedial arthritis or medial osteonecrosis (ON) of the knee [1, 2]. The Oxford mobile bearing has some advantages, including a low rate of bearing wear, favorable longevity, and minimization of shear stress at the bone–implant interfaces [1, 3]. However, Kennedy et al. [4] reported that elderly patients (aged ≥75 years) had significantly lower postoperative clinical scores than younger patients treated with Oxford UKA. Furthermore, Siman et al. [5] compared UKA with total knee arthroplasty (TKA) in elderly patients (aged ≥75 years) with isolated medial compartmental arthritis and reported that the postoperative Knee Society Score of UKA was unfortunately equivalent to that of TKA. In contrast, some studies reported that the clinical outcomes of UKA were superior to those of TKA over wide age ranges, including elderly patients [6–8]. Therefore, the clinical results after UKA in elderly patients could be inferior to those in nonelderly patients. However, no study has yet investigated the reasons for the difference in clinical outcomes between elderly and nonelderly patients treated with UKA.

Recently, some studies focused on intraoperative kinematics measured by the navigation system or specific devices in UKA and reported that intraoperative kinematics might be related to clinical outcomes [9–11]. Moreover, the relationship between intraoperative rotational kinematics and postoperative clinical results of TKA has been established by some previous studies [12–15]. Specifically, Ishida et al. [13, 14] focused on the relationship between intraoperative tibial rotation relative to the femur and postoperative clinical results in posterior stabilized TKA and reported that intraoperative tibial internal rotation patterns affected the postoperative knee flexion angle. Moreover, Kamenaga et al. [15] demonstrated that the degree of tibial internal rotation from a flexion angle of 60° to 135° had a positive relationship with the postoperative flexion angle. These studies help surgeons predict and modify the clinical outcomes of TKA from the intraoperative rotational kinematics. Especially in UKA, Inui et al. [9] reported that the femorotibial rotational mismatch was related to lower postoperative clinical outcomes. Therefore, based on these previous studies, intraoperative rotational kinematics in UKA could be the reason for the difference in clinical outcomes between elderly and nonelderly patients. However, there is no information regarding the age difference in intraoperative kinematics, and obviously, we are unaware of the age difference in the relationship between intraoperative rotational kinematics and postoperative clinical results.

We hypothesized that there would be an age difference in intraoperative rotational kinematics and, moreover, the intraoperative rotational kinematics would have an impact on the clinical results in all elderly patients. Therefore, we conducted this study to explore the age difference in intraoperative rotational kinematics and also in the relationship between intraoperative rotational kinematics and postoperative clinical outcomes.

## Materials and methods

We conducted a retrospective review of UKA procedures performed at our institution. Of 196 primary medial mobile-bearing Oxford UKA (Zimmer Biomet Ltd., Swindon, UK) procedures performed consecutively between September 2012 and May 2018 using an image-free navigation system (Precision N; Stryker Orthopedics, Mahwah, NJ, USA), 111 knees had available intraoperative measures of rotational kinematics and were included in this study. The other 85 knees underwent conventional UKA without the navigation or UKA using the portable navigation system. The accuracy of this navigation system has been confirmed in our previous studies [16, 17]. Surgery was performed for isolated anteromedial osteoarthritis (OA) with bone-on-bone articulation or ON of the medial femoral condyle. Both the cruciate and collateral ligaments were functionally intact in all patients. The surgical indication was made as defined by the Oxford Group [18]. We divided 111 knees who underwent Oxford UKA into two groups: elderly (aged ≥75 years; 48 knees) and nonelderly (aged < 75 years; 63 knees) based on the previous cut-off point [4] and, we compared several parameters, that were shown below, between the two groups to explore the age difference.

### Surgical procedure

All surgeries were performed using a minimally invasive approach to comply with the Oxford Group recommendations [18]. The registered anatomical landmarks comprised the center of the femoral head, the distal femur, the proximal tibia and the ankle, the femoral temporary antero-posterior (AP) axis, and the temporary tibial AP axis. The temporal femoral AP axis on the navigation system was defined as the Whiteside axis, and the temporal tibial AP axis was determined as the Akagi’s line connecting the middle of the posterior cruciate ligament to the medial border of the patellar tendon [19]. The center of the femoral head was determined by rotating the femur by rotational calculations. The center of the ankle was represented by a 44–56% medial-to-lateral ratio along the transmalleolar axis. After setting the tibial cutting guide, the alignment of the cutting guide was measured using the navigation system. Thereafter, a tibial vertical cut was made at the medial edge of the anterior cruciate ligament insertion on the tibia with reference to the hip center, the anterior superior iliac spine, and Shakespeare’s line [20]. Then, a tibial cut was made. Femoral drilling was performed using a device to facilitate reproducible femoral implantation [21]. After completing these procedures, we performed the same gap-balancing procedure between knee flexion and extension and a modified keel cutting method, as reported previously [22].

### Intraoperative evaluation

**Fig. 1:**
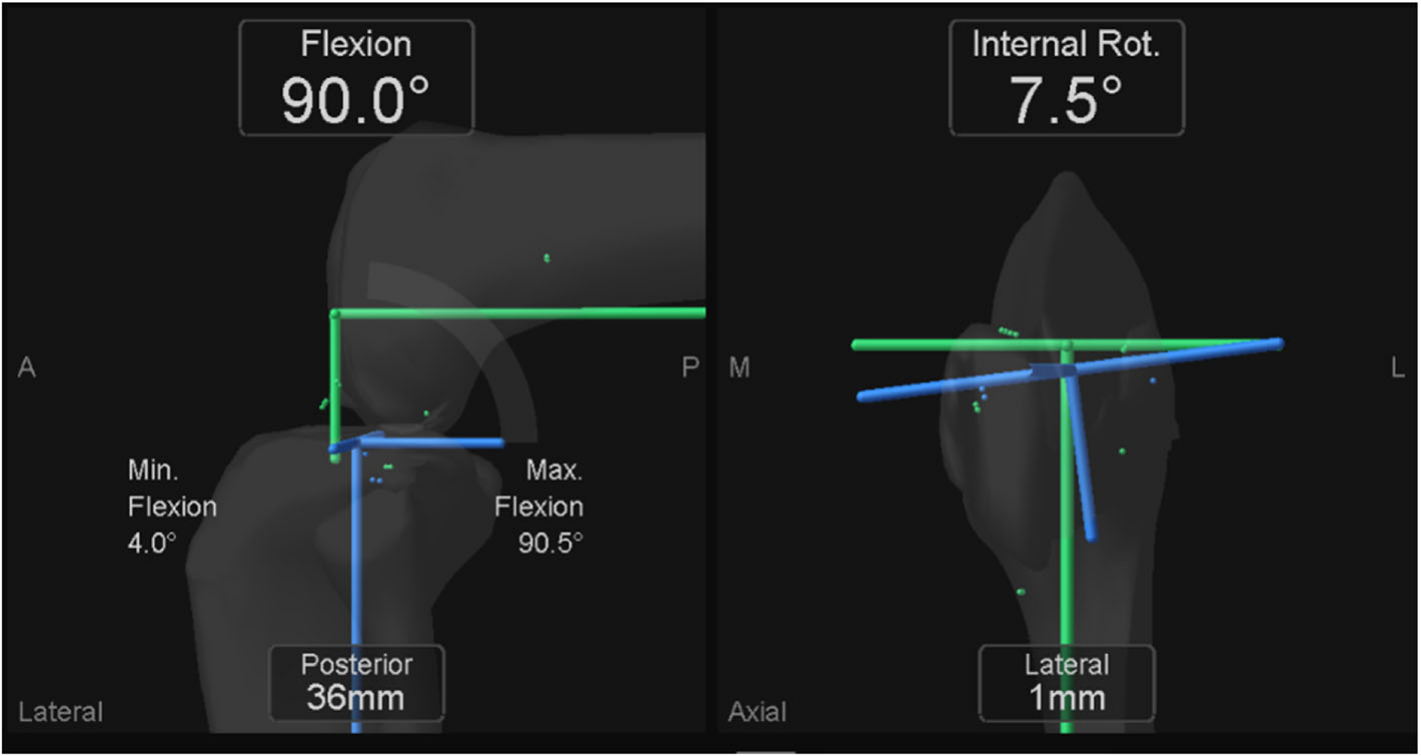
Intraoperative tibial rotation relative to the femur evaluated by the image-free navigation system. This image showed that the tibial antero-posterior axis internally rotated 7.5° relative to the femoral antero-posterior axis at 90° knee flexion

Intraoperative tibial internal rotations relative to the femur were evaluated for each of the 111 patients using the image-free navigation according to previous method [9]. After performing all of the osteotomy necessary for the procedure using the navigation system, we cancelled the temporary AP axis and re-registered the AP axis of the femur and tibia for the measurement of tibial rotation between the femoral AP axis and tibial AP axis of the components. Because we thought it is difficult to identify the rotational axis accurately, in particular, the Akagi’s line in the narrow operating field during UKA, we re-registered the AP axis and evaluated the rotational angle not between the femur bone and tibia bone but between the femur component and tibial component. In short, the new AP axis of the femur was aimed along the line connecting two peg holes, which is the rotational axis of the Oxford femoral component, and the new AP axis of the tibia was aimed along the lateral wall of the tibial tray. The tibial internal rotation was defined the rotational angle of the new tibial AP axis relative to the new femoral AP axis in axial plane and, the angle calculated automatically in the navigation system (Fig. 1). The tibial rotational angle relative to the femur at the maximum extension was set to 0°, considering a registration error of image-free navigation. Tibial rotational angles were evaluated during the knee flexion cycles from the maximum extension to the maximum flexion (flexion angles at maximum extension, 30°, 60°, 90°, and maximum flexion). From among the rotational kinematics data, we presented the following three parameters in the present study, considering the data of previous studies as well [3–6]: (1) the rotational angle as the entire knee flexion from the maximum extension to the maximum flexion; (2) the rotational angle as the first half of knee flexion from the maximum extension to 90° knee flexion; and (3) the rotational angle as the second half of knee flexion from 90° knee flexion to the maximum flexion. Tibial internal rotation relative to the femur was defined as a positive value.

### Clinical evaluation and radiographic evaluation

The preoperative and postoperative Knee Society Functional Score (KSFS) was documented, and knee extension and flexion angles were measured using a two-arm goniometer, with the patient in the supine position, at both the preoperative and the 2-year postoperative follow-up visit. Preoperative and postoperative subjective scores were also evaluated using the validated version of the Knee Injury and Osteoarthritis Outcome Score (KOOS), which is a self-reported questionnaire consisting of 42 items addressing five separately analyzed subscales of pain, symptoms, and activities of daily living (ADL) for physical function, sport recreation function, and knee-related quality of life (QOL). Each of the five scores is calculated as the sum of the included items and converted into a 0- to 100-point scale, with 0 points representing extreme knee problems and 100 points representing no knee problems [23, 24]. The preoperative KOOS was not available for the 19 early cases (8 elderly and 11 nonelderly patients). Regarding preoperative and postoperative radiographic evaluation, a femorotibial angle (FTA) in the frontal plane was measured with full-length standing plain radiography.

### Statistical analyses

All statistical analyses were conducted using the SPSS v.25.0 statistical software (IBM Corp., Armonk, NY). Preoperative and postoperative parameters (range of motion, KSFS, FTA, and KOOS) were compared between the two groups using the unpaired *t*-test. Linear regression analysis was performed to evaluate the correlation between the clinical outcomes and the tibial rotational angle relative to the femur. Regarding the comparison of the intraoperative tibial rotation using the unpaired *t*-test, a statistical power analysis was performed after the study using G power 3 [25] and an α power of 0.53. All significance tests were two-tailed, and a significance level of *p* < 0.05 was used for all tests. Interclass and intraclass coefficient values of the intraoperative tibial rotational angle relative to the femur evaluated using the navigation system were > 0.80, indicating excellent reliability.

## Results

**Fig. 2:**
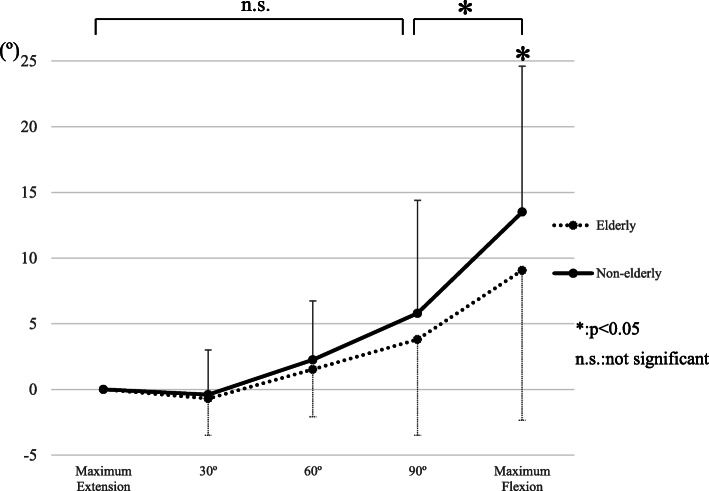
Intraoperative tibial rotation relative to the femur in elderly (aged ≥75 years) and nonelderly patients (aged < 75 years). Intraoperative tibial rotation was defined as the angle between the tibial anteroposterior (AP) axis and the femoral AP axis in axial plane and, tibial internal rotation relative to the femur was defined a plus value. The tibial internal rotational angles during the entire knee flexion and from 90° to full flexion were significantly larger in nonelderly patients

Preoperative demographic data revealed that only the body mass index was significantly higher in the nonelderly group, whereas no significant differences were observed in other demographic parameters, except age (Table 1). The intraoperative tibial internal rotational angles relative to the femur during the entire knee flexion were 9.0° in the elderly group and 13.5° in the nonelderly group, and the tibial internal rotational angles from extension to 90° flexion and from 90° flexion to full flexion were 3.8° and 4.5° in the elderly group and 5.8° and 7.7° in the nonelderly group, respectively. The tibial internal rotational angles during the entire knee flexion and from 90° to full flexion were significantly larger in nonelderly patients (Table 2, Fig. 2). The postoperative KSFS and ADL, sports subscales in the KOOS, which were contained as the activity scores, were significantly higher in the nonelderly group; however, there were no significant differences in other postoperative clinical outcomes (Table 3). In the nonelderly group, the intraoperative tibial internal rotation during the entire knee flexion correlated positively with the pain subscale in the KOOS, and the intraoperative tibial rotation from 90° to full flexion correlated positively with the pain and QOL subscales in the KOOS. However, in the elderly group, no correlation was detected between the intraoperative tibial rotation and postoperative clinical outcomes (Tables 4, 5).

**Table 1:** Preoperative demographics between elderly patients (aged ≥75 years) and nonelderly patients (aged < 75 years)

	Elderly	Non-elderly	*P* value
Number of knees	48	63	
Gender (female/male)	29/19	46/17	0.219
Diagnosis (OA/ON)	42/6	49/14	0.220
Age (years)	79.3 (3.1)	66.6 (5.6)	< 0.001*
Body mass index (kg/m^2^)	24.3 (3.4)	25.8 (4.1)	0.028*
Maximum extension (°)	4.6 (3.4)	3.5 (4.3)	0.159
Maximum flexion (°)	131.0 (6.8)	131.4 (6.7)	0.963
Knee society functional score	54.1 (13.3)	58.2 (14.7)	0.135
Femorotibial angle (°) in X-ray	182.9 (3.8)	182.3 (4.3)	0.426
KOOS (knees)	40	52	
Pain	52.2 (15.1)	47.8 (15.5)	0.185
Symptom	59.6 (19.4)	57.9 (17.8)	0.666
ADL	60.0 (13.0)	59.4 (16.4)	0.837
Sports	22.7 (17.4)	22.6 (17.9)	0.981
QOL	27.1(15.6)	29.5 (15.8)	0.477

Data presented as Average (Standard deviation)

*statistically significant (*p* < 0.05)

*OA* osteoarthritis, *ON* osteonecrosis, *KOOS* knee injury and osteoarthritis outcome score, *ADL* activities of daily living, *QOL* quality of life

**Table 2:** Intraoperative tibial rotation relative to the femur in elderly and nonelderly patients and comparison between elderly patients (aged ≥75 years) and nonelderly patients (aged < 75 years)

Tibial internal rotation angle	Elderly	Non-elderly	*P* value
Extension~max Flexion	9.0 (11.4)	13.5 (11.1)	0.041*
Extension~ 90°	3.8 (7.3)	5.8 (8.7)	0.215
90° ~ max Flexion	5.2 (5.4)	7.7 (4.8)	0.013*

Data presented as Average (Standard deviation)

*statistically significant (*p* < 0.05)

*max* maximum

**Table 3:** Postoperative clinical results between elderly patients (aged ≥75 years) and nonelderly patients (aged < 75 years)

	Elderly	Non-elderly	*P* value
Maximum extension (°)	1.6 (2.7)	1.1 (2.0)	0.171
Maximum flexion (°)	128.7 (7.9)	131.0 (8.1)	0.142
KOOS			
Pain	85.4 (11.9)	88.8 (11.9)	0.143
Symptom	85.4 (11.8)	86.0 (11.2)	0.800
ADL	82.0 (12.9)	88.3 (11.8)	0.010*
Sports	52.3 (25.3)	63.7 (25.4)	0.022*
QOL	65.4 (21.3)	71.6 (18.3)	0.104
Knee society functional score	78.8 (16.0)	88.1 (14.0)	0.002*
Femorotibial angle (°) in X-ray	178.1 (3.1)	177.2 (3.0)	0.137

Data presented as Average (Standard deviation)

*statistically significant (p < 0.05)

*KOOS* knee injury and osteoarthritis outcome score, *ADL* activities of daily living, *QOL* quality of life

**Table 4:** Correlation coefficients between intraoperative tibial internal rotation and postoperative clinical scores in elderly patients (aged ≥75 years)

	Tibial internal rotation		
	Ex~full Flex	Ex~ 90°	90° ~ full Flex
KOOS			
Pain	−0.04	−0.24	0.04
Symptom	−0.09	−0.25	0.03
ADL	−0.04	− 0.15	0.05
Sports	0.01	−0.02	0.06
QOL	− 0.05	− 0.17	0.03
Knee Society functional score	−0.08	0.16	−0.15
Knee flexion angle	0.01	−0.21	0.02

Data presented as correlation coefficient

*Correlations are statistically significant (*p* < 0.05)

*KOOS* knee injury and osteoarthritis outcome score, *ADL* activities of daily living, *QOL* quality of life

**Table 5:** Correlation coefficients between intraoperative tibial internal rotation and postoperative clinical scores in nonelderly patients (aged < 75 years)

	Tibial internal rotation		
	Ex~full Flex	Ex~ 90°	90° ~ full Flex
KOOS			
Pain	0.27*	0.15	0.37*
Symptom	−0.03	−0.09	0.07
ADL	0.02	−0.05	0.14
Sports	0.01	−0.05	0.12
QOL	0.18	0.06	0.31*
Knee Society functional score	0.06	0.06	0.02
Knee flexion angle	−0.04	−0.08	0.06

Data presented as correlation coefficient

*Correlations are statistically significant (p < 0.05)

*KOOS* knee injury and osteoarthritis outcome score, *ADL* activities of daily living, *QOL* quality of life

## Discussion

The most important findings in this study on UKA were that the intraoperative tibial rotation during knee flexion was larger in nonelderly patients than in elderly patients, and the intraoperative tibial internal rotation had a positive influence on the postoperative clinical outcomes only in nonelderly patients.

Regarding the kinematics in Oxford UKA, Kono et al. [26] compared the in vivo preoperative and postoperative kinematics in Oxford UKA using fluoroscopic 2D-3D registration technique and reported that the tibiofemoral preoperative rotational kinematics was maintained postoperatively after the Oxford mobile-bearing UKA procedure. In addition, Oxford mobile-bearing UKA has been suggested to be superior to fixed-bearing UKA in restoring the normal tibiofemoral biomechanics [27–29]. However, no study has investigated the age difference in perioperative kinematics in UKA. To our knowledge, our study is the first to demonstrate the age difference in intraoperative tibiofemoral rotational kinematics during knee flexion, reporting that the tibial internal rotation was larger in nonelderly patients. In normal knees, the tibial internal rotation during knee flexion has been reported to be a “medial pivot motion,” and this tibial internal rotation could be generated by the four-bar linkage of both cruciate and collateral ligaments [30–32]. Therefore, it was believed that the difference in internal rotation between two groups could be the condition of ligaments and related soft tissues. However, the normal axial rotation decreased proportionately with the progression of medial-grade OA [33]. Therefore, we compared the grade of OA between the elderly and nonelderly patients and observed that the preoperative bone varus deformity in the X-ray was not different between the two patient groups. In addition, this finding supported our theory that the difference in the tibial internal rotation was derived from the quality of related soft tissues.

Nevertheless, UKA has become a standard surgery for not only middle-aged patients but also elderly patients [4, 5, 34, 35]. Iacono et al. [35] evaluated the mid- to long-term clinical and radiographic results after Oxford mobile-bearing UKA in elderly patients (aged ≥75 years) and reported that their postoperative range of motion, Oxford Knee Score, Knee Society Score, and WOMAC score were satisfactory and the tibial component alignment was still maintained at the 9-year follow-up. They concluded that UKA was a viable option to treat medial OA even for elderly patients in terms of pain relief and activity scores. In contrast, Siman et al. [5] reported that in elderly patients (aged ≥75 years), UKA could not result in a superior Knee Society Score to that in patients undergoing TKA, although patients undergoing UKA had shorter hospital stay, shorter operative time, and lower estimated blood loss than patients undergoing TKA. Furthermore, Kennedy et al. [4] reported that the Oxford Knee Score after UKA in elderly patients was significantly lower than that in younger patients. Nevertheless, these studies did not distinguish clinical pain relief and clinical recovery of activity postoperatively, because elderly patients with lower muscle strength could have lower potential in recovery of activity than nonelderly patients who have higher muscle strength. The difference between TKA and UKA could disappear because of the lower activity recovery in elderly patients. Therefore, it is necessary to evaluate postoperative clinical results separately with the pain relief and activity components when evaluating elderly patients. From this viewpoint, the present study is meaningful and our results are reasonable, that is, pain relief and flexion angle after UKA were achieved in both elderly and nonelderly patients, and the activity scores after UKA were greater in nonelderly patients than in elderly patients. However, Fabre-Aubrespy et al. [34] evaluated the clinical outcomes of elderly patients (aged ≥75 years) who underwent fixed-bearing UKA and distinguished pain relief scores from activity scores. They reported that not only pain relief scores (Pain and Symptom in the KOOS, Knee Society Score) but also the recovery of activity scores (NKFS and ADL in KOOS) in their study were better for elderly patients treated with UKA than for elderly patients treated with TKA. Their results confirmed that the activity score after UKA in elderly patients was less than that in nonelderly patients, but more than that in elderly patients undergoing TKA, thereby indicating that UKA was a viable surgical option even for elderly patients in terms of pain relief and recovery of activity.

Importantly, several recent studies have emphasized the extent of intraoperative tibial rotation in TKA [14, 15, 36], and the majority of them have demonstrated a relationship between the tibial internal rotational angle and the postoperative flexion angle. Furthermore, the majority of studies have focused on the tibial internal rotational angle during the latter half of knee flexion [14, 15, 36]. In the present study, the intraoperative tibial internal rotation during the entire knee flexion correlated positively with the pain subscale in the KOOS, and the internal rotational angle from 90° to full flexion correlated positively with the pain and QOL subscales in the KOOS only in nonelderly patients. The primary reason for the correlation between internal rotation during the latter half of knee flexion and clinical outcomes is believed to be the medial pivot motion of UKA. Kono et al. [37] described the postoperative non-weight-bearing kinematics of UKA in 24 functionally well knees and reported the tibial internal rotation during the latter half of knee flexion. In their study, the lateral contact point constantly moved posteriorly, whereas the medial contact point moved only slightly, especially during the latter half of knee flexion. This would be the so-called medial pivot motion associated with Oxford UKA in a non-weight-bearing position. Non-weight-bearing kinematics of the knee after surgery is expected to be similar to intraoperative kinematics [38]. We could not evaluate the accurate anterior–posterior position of the femur relative to the tibia, in particular, for each component from our navigation data, and it was difficult to distinguish whether intraoperative tibial internal rotation was the central pivot motion or the medial pivot motion. Nevertheless, our results in nonelderly patients appear remarkably similar to previous reports that showed that the medial pivot motion during the operation correlated with better subjective scores in TKA [39]. Therefore, we believed that in UKA for nonelderly patients, the intraoperative medial pivot motion during the latter half of knee flexion resulted in excellent postoperative KOOSs. However, we could not identify any correlations between the intraoperative tibial rotation and clinical results in elderly patients. These patients had smaller intraoperative tibial internal rotation than nonelderly patients, but their postoperative patient-reported outcomes, except for activity scales, were not different from those of nonelderly patients. This implied that the relationship between kinematics and clinical outcomes in elderly patients was weaker than that in nonelderly patients, and multiple factors, such as painful duration, muscle strength, and lumbar disorders, could influence postoperative patient-reported outcomes in elderly patients. Nonetheless, we could not find any correlation between intraoperative tibial internal rotation and postoperative knee flexion angle in both our study groups, unlike that in previous studies on TKA [14, 15, 36]. The reason for this difference between TKA and UKA is that the postoperative flexion angle after UKA was relatively good in almost all patients. Therefore, the difference in intraoperative rotation did not affect the postoperative knee flexion angle.

This study has certain limitations. First, the patients were divided into two groups based on the age of 75 years; therefore, all those aged < 75 years were grouped into one group. This relatively young group contained patients aged 54–74 years, and differences in clinical outcomes and intraoperative kinematics would certainly exist within this group. Therefore, a further investigation is planned with a larger cohort. Second, the follow-up period was relatively short. However, we experienced only a few problems related to loosening, polyethylene wear, or breakage of Oxford UKA. Hence, a larger sample size with a longer follow-up period is required. Third, this study was a retrospective and single center study. Fourth, the intraoperative kinematics was evaluated by only one kind of navigation system. Fifth, although the KOOS is a valid, reliable, and responsive outcome measure in patients with knee arthroplasty, minimal clinically important differences in the KOOS were not apparent. Sixth, the procedures were performed by five knee surgeons, which raises the possibility of interobserver bias. Seventh, we did not evaluate the alignment of the component. Eighth, preoperative KOOS data were not available for all patients; however, *p* values in the preoperative KOOS were relatively high.

Regarding clinical relevance of this study, this study showed that rotational kinematics itself and its clinical relevance were different according to age, therefore, in UKA, targeted kinematics, alignment and soft-tissue balance could be different among ages. Surgical techniques and postoperative alignments to retain the tibial internal rotation during knee flexion could be more important especially for nonelderly patients. Further study should be needed in a larger sample size.

## Conclusion

Intraoperative rotational kinematics and its influence on clinical outcomes were different between elderly and nonelderly patients, and tibial internal rotation could be a more important factor for successful UKA in nonelderly patients. Postoperative clinical outcomes, except for activity parameters, were not significantly different between the two groups.

## Data Availability

The datasets used during the current study are available from the corresponding author on reasonable request.

## Abbreviations

UKA: Unicompartmental knee arthroplasty
TKA: Total knee arthroplasty
OA: Osteoarthritis
AP: Antero-posterior
KOOS: Knee injury and osteoarthritis outcome score
ADL: Activities of daily living
KSFS: Knee society functional score

## References

[1] Pandit H, Hamilton TW, Jenkins C, Mellon SJ, Dodd CA, Murray DW (2015). The clinical outcome of minimally invasive phase 3 Oxford unicompartmental knee arthroplasty: a 15-year follow-up of 1000 UKAs. J Bone Joint Surg Br.

[2] Yoshida K, Tada M, Yoshida H, Takei S, Fukuoka S, Nakamura H (2013). Oxford phase 3 unicompartmental knee arthroplasty in Japan--clinical results in greater than one thousand cases over ten years. J Arthroplast.

[3] Kendrick BJ, Simpson DJ, Kaptein BL, Valstar ER, Gill HS, Murray DW, Price AJ (2011). Polyethylene wear of mobile-bearing unicompartmental knee replacement at 20 years. J Bone Joint Surg Br.

[4] Kennedy JA, Matharu GS, Hamilton TW, Mellon SJ, Murray DW (2018). Age and outcomes of medial meniscal-bearing Unicompartmental knee Arthroplasty. J Arthroplast.

[5] Siman H, Kamath AF, Carrillo N, Harmsen WS, Pagnano MW, Sierra RJ (2017). Unicompartmental knee Arthroplasty vs Total knee Arthroplasty for medial compartment arthritis in patients older than 75 years: comparable reoperation, revision, and complication rates. J Arthroplast.

[6] Liebensteiner M, Köglberger P, Ruzicka A, Giesinger JM, Oberaigner W, Krismer M (2020). Unicondylar vs. total knee arthroplasty in medial osteoarthritis: a retrospective analysis of registry data and functional outcome. Arch Orthop Trauma Surg.

[7] Blevins JL, Carroll KM, Burger JA, Pearle AD, Bostrom MP, Haas SB, Sculco TP, Jerabek SA, Mayman DJ (2020). Postoperative outcomes of total knee arthroplasty compared to unicompartmental knee arthroplasty: a matched comparison. Knee.

[8] Kleeblad LJ, van der List JP, Zuiderbaan HA, Pearle AD (2018). Larger range of motion and increased return to activity, but higher revision rates following unicompartmental versus total knee arthroplasty in patients under 65: a systematic review. Knee Surg Sports Traumatol Arthrosc.

[9] Inui H, Taketomi S, Yamagami R, Kono K, Kawaguchi K, Takagi K, Kage T, Tanaka S (2020). Femorotibial rotational mismatch of the Oxford unicompartmental knee in the flexion position is a risk for poor outcomes. Knee.

[10] Kawaguchi K, Inui H, Taketomi S, Yamagami R, Nakazato K, Shirakawa N, Tanaka S (2019). Intraoperative mobile-bearing movement in Oxford unicompartmental knee arthroplasty. Knee Surg Sports Traumatol Arthrosc.

[11] Suzuki T, Ryu K, Kojima K, Oikawa H, Saito S, Nagaoka M (2019). The effect of posterior Tibial slope on joint gap and range of knee motion in Mobile-bearing Unicompartmental knee Arthroplasty. J Arthroplast.

[12] Inokuchi T, Ishida K, Takayama K, Shibanuma N, Hayashi S, Kurosaka M, Kuroda R, Matsumoto T (2019). Intraoperative posterior movement of the tibia at 90° of flexion predicts worse postoperative flexion angles in cruciate-substituting total knee arthroplasty. Knee Surg Sports Traumatol Arthrosc.

[13] Ishida K, Shibanuma N, Matsumoto T, Sasaki H, Takayama K, Hiroshima Y, Kuroda R, Kurosaka M (2016). Navigation-based tibial rotation at 90 degrees of flexion is associated with better range of motion in navigated total knee arthroplasty. Knee Surg Sports Traumatol.

[14] Ishida K, Shibanuma N, Matsumoto T, Sasaki H, Takayama K, Matsuzaki T, Tei K, Kuroda R, Kurosaka M (2016). Navigation-based femorotibial rotation pattern correlated with flexion angle after total knee arthroplasty. Knee Surg Sports Traumatol Arthrosc.

[15] Kamenaga T, Takayama K, Ishida K, Muratsu H, Hayashi S, Hashimoto S, Kuroda Y, Tsubosaka M, Takashima Y, Matsushita T, Niikura T, Kuroda R, Matsumoto T (2019). Medial knee stability at flexion increases tibial internal rotation and knee flexion angle after posterior-stabilized total knee arthroplasty. Clin Biomech.

[16] Inui H, Taketomi S, Nakamura K, Takei S, Takeda H, Tanaka S, Nakagawa T (2013). Influence of navigation system updates on total knee arthroplasty. BMC Sports Sci Med Rehabil.

[17] Kawaguchi K, Inui H, Taketomi S, Yamagami R, Nakazato K, Tanaka S (2019). Intraoperative Tibial Anteroposterior Axis could not be replicated after Tibial osteotomy in Total knee Arthroplasty. J Arthroplast.

[18] Goodfellow JW OCJ, Pandit H, Dodd C, Murray D. Unicompartmental arthroplasty with the Oxford knee. 2nd ed. Goodfellow Publishers. United Kingdom: Oxford University Press; 2015. p. 69–89.

[19] Akagi M, Oh M, Nonaka T, Tsujimoto H, Asano T, Hamanishi C (2004). An anteroposterior axis of the tibia for total knee arthroplasty. Clin Orthop Relat Res.

[20] Shakespeare D, Ledger M, Kinzel V (2005). The influence of the tibial sagittal cut on component position in the Oxford knee. Knee.

[21] Inui H, Taketomi S, Yamagami R, Sanada T, Shirakawa N, Tanaka S (2016). Impingement of the Mobile bearing on the Lateral Wall of the Tibial tray in Unicompartmental knee Arthroplasty. J Arthroplast.

[22] Inui H, Taketomi S, Yamagami R, Sanada T, Tanaka S (2016). Twice cutting method reduces tibial cutting error in unicompartmental knee arthroplasty. Knee.

[23] Nakamura N, Takeuchi R, Sawaguchi T, Ishikawa H, Saito T, Goldhahn S (2011). Cross-cultural adaptation and validation of the Japanese knee injury and osteoarthritis outcome score (KOOS). J Orthop Sci.

[24] Roos EM, Lohmander LS (2003). The knee injury and osteoarthritis outcome score (KOOS): from joint injury to osteoarthritis. Health Qual Life Outcomes.

[25] Faul F, Erdfelder E, Buchner A, Lang AG (2009). Statistical power analyses using G*power 3.1: tests for correlation and regression analyses. Behav Res Methods.

[26] Kono K, Inui H, Tomita T, Yamazaki T, Taketomi S, Yamagami R, Kawaguchi K, Sugamoto K, Tanaka S (2020). In vivo kinematic comparison before and after mobile-bearing unicompartmental knee arthroplasty during high-flexion activities. Knee.

[27] Gleeson RE, Evans R, Ackroyd CE, Webb J, Newman JH (2004). Fixed or mobile bearing unicompartmental knee replacement? A comparative cohort study. Knee.

[28] Li MG, Yao F, Joss B, Ioppolo J, Nivbrant B, Wood D (2006). Mobile vs. fixed bearing unicondylar knee arthroplasty: a randomized study on short term clinical outcomes and knee kinematics. Knee.

[29] Hing CB, Davies L, Donell ST, Smith TO (2009). Fixed versus mobile bearing unicompartmental knee replacement: a meta-analysis. Orthop Traumatol Surg Res.

[30] Ren S, Yu Y, Shi H, Miao X, Jiang Y, Liang Z, Hu X, Huang H, Ao Y (2018). Three dimensional knee kinematics and kinetics in ACL-deficient patients with and without medial meniscus posterior horn tear during level walking. Gait Posture.

[31] Murayama T, Sato T, Watanabe S, Kobayashi K, Tanifuji O, Mochizuki T, Yamagiwa H, Koga Y, Omori G, Endo N (2016). Three-dimensional in vivo dynamic motion analysis of anterior cruciate ligament-deficient knees during squatting using geometric center axis of the femur. J Orthop Sci.

[32] Matsumoto H, Seedhom BB (1993). Rotation of the tibia in the normal and ligament-deficient knee. A study using biplanar photography. Proceedings of the Institution of Mechanical Engineers Part H. J Eng Med.

[33] Nagao N, Tachibana T, Mizuno K (1998). The rotational angle in osteoarthritic knees. Int Orthop.

[34] Fabre-Aubrespy M, Ollivier M, Pesenti S, Parratte S, Argenson JN (2016). Unicompartmental knee Arthroplasty in patients older than 75 results in better clinical outcomes and similar survivorship compared to Total knee Arthroplasty. A Matched Controlled Study. J Arthroplasty.

[35] Iacono F, Raspugli GF, Akkawi I, Bruni D, Filardo G, Budeyri A, Bragonzoni L, Presti ML, Bonanzinga T, Marcacci M (2016). Unicompartmental knee arthroplasty in patients over 75 years: a definitive solution?. Arch Orthop Trauma Surg.

[36] Matsuzaki T, Matsumoto T, Muratsu H, Ishida K, Takayama K, Nagai K, Nakano N, Nishida K, Kuroda R, Kurosaka M (2017). The contribution of intraoperative medial compartment stability to post-operative knee flexion angle in unicompartmental knee arthroplasty. Knee Surg Sports Traumatol Arthrosc.

[37] Kono K, Inui H, Tomita T, Yamazaki T, Taketomi S, Yamagami R, et al. Weight-bearing status affects in vivo kinematics following mobile-bearing unicompartmental knee arthroplasty. Knee Surg Sports Traumatol Arthrosc. 2020;(3):718–24. 10.1007/s00167-020-05893-x. 10.1007/s00167-020-05893-x 32055876

[38] Wada K, Mikami H, Hamada D, Yamazaki T, Tomita T, Sairyo K (2017). Can intraoperative kinematic analysis predict postoperative kinematics following total knee arthroplasty? A preliminary. J Med Investig.

[39] Nishio Y, Onodera T, Kasahara Y, Takahashi D, Iwasaki N, Majima T (2014). Intraoperative medial pivot affects deep knee flexion angle and patient-reported outcomes after total knee arthroplasty. J Arthroplast.

